# Modulation of the Liver Protein Carbonylome by the Combined Effect of Marine Omega-3 PUFAs and Grape Polyphenols Supplementation in Rats Fed an Obesogenic High Fat and High Sucrose Diet

**DOI:** 10.3390/md18010034

**Published:** 2019-12-30

**Authors:** Lucía Méndez, Silvia Muñoz, Bernat Miralles-Pérez, Maria Rosa Nogués, Sara Ramos-Romero, Josep Lluis Torres, Isabel Medina

**Affiliations:** 1 Instituto de Investigaciones Marinas-Consejo Superior de Investigaciones Científicas (IIM-CSIC), Eduardo Cabello 6, E-36208 Vigo, Spain; silviam@iim.csic.es (S.M.); medina@iim.csic.es (I.M.); 2 Unidad de Farmacología, Facultad de Medicina, Universidad Rovira i Virgili, Sant Llorenç 21, E-43201 Reus, Spainmariarosa.nogues@urv.cat (M.R.N.); 3 Instituto de Química Avanzada de Cataluña-Consejo Superior de Investigaciones Científicas (IQAC-CSIC), Jordi Girona 18-26, E-08034 Barcelona, Spain; sara.ramos@iqac.csic.es (S.R.-R.); joseplluis.torres@iqac.csic.es (J.L.T.); 4 Departamento de Biología Celular, Fisiología e Inmunología, Facultad de Biología, Universitad de Barcelona, Diagonal 643, E-08028 Barcelona, Spain

**Keywords:** fish oils, grape polyphenols, protein carbonylation, high-fat, high sucrose diet, omega-3 PUFAs, diet-induced obesity

## Abstract

Diet-induced obesity has been linked to metabolic disorders such as cardiovascular diseases and type 2 diabetes. A factor linking diet to metabolic disorders is oxidative stress, which can damage biomolecules, especially proteins. The present study was designed to investigate the effect of marine omega-3 polyunsaturated fatty acids (PUFAs) (eicosapentaenoic acid (EPA) and docosahexaenoic acid (DHA)) and their combination with grape seed polyphenols (GSE) on carbonyl-modified proteins from plasma and liver in Wistar Kyoto rats fed an obesogenic diet, namely high-fat and high-sucrose (HFHS) diet. A proteomics approach consisting of fluorescein 5-thiosemicarbazide (FTSC) labelling of protein carbonyls, visualization of FTSC-labelled protein on 1-DE or 2-DE gels, and protein identification by MS/MS was used for the protein oxidation assessment. Results showed the efficiency of the combination of both bioactive compounds in decreasing the total protein carbonylation induced by HFHS diet in both plasma and liver. The analysis of carbonylated protein targets, also referred to as the ‘carbonylome’, revealed an individual response of liver proteins to supplements and a modulatory effect on specific metabolic pathways and processes due to, at least in part, the control exerted by the supplements on the liver protein carbonylome. This investigation highlights the additive effect of dietary fish oils and grape seed polyphenols in modulating in vivo oxidative damage of proteins induced by the consumption of HFHS diets.

## 1. Introduction

The consumption of high fat and high sucrose diets leads to obesity, which represents a severe worldwide public health concern in terms of contribution to human diseases. Obesity increases the likelihood of developing other serious conditions, including cardiovascular diseases and type 2 diabetes [1]. Understanding the molecular mechanisms behind the development of these obesity-related diseases is critical for the assessment of more appropriate and successful prevention/palliation strategies against metabolic disorders. 

Oxidative stress is often defined as imbalance between pro-oxidants and antioxidants [2] or, more recently, as disturbance of redox signaling and control [3]. Chronic oxidative stress results in increased levels of oxidized proteins. This fact can be enough to trigger cellular dysfunction since almost the whole metabolism relies on proteins to execute key cellular processes [4]. Proteins can be modified by several oxidative modifications but protein carbonylation is considered a major hallmark of oxidative damage [5]. Protein carbonylation is the consequence of a non-enzymatic phenomenon that yields a reactive carbonyl moiety (aldehyde, ketone, or lactam) in a protein and leads protein damage. There are four different mechanisms to form protein-bound carbonyls: (a) By metal-catalyzed oxidation of the side chains of lysine, proline, arginine, and tyrosine; (b) by direct oxidation of tryptophan; (c) by glycoxidation―protein-bound carbonyls as adducts of advanced glycation products (AGEs)—on lysine and arginine; and (d) by lipid peroxidation products which react with cysteine, histidine, and lysine (alkenals Michael adducts) [6]. Proteins are selectively carbonylated in vivo, depending on amino acid sequence (nucleophilic amino acids and surrounding sequence) [7], but also tridimensional conformation, cellular location, and function [8], among others. Several pieces of information have linked protein carbonylation to obesity and derived diseases [9]. It has been reported that overnutrition induces extensive carbonylation of GLUT4 in adipose tissue, which leads to protein activity loss, and then insulin resistance [10]. 

The consumption of current western diets rich in fat and sugar induces metabolic alterations [1] but also increases oxidative stress and specifically, protein carbonylation [11]. Effective dietary strategies to prevent and counteract the development of these alterations include the use of natural bioactive compounds for maintaining redox homeostasis [12]. It has been largely accepted that consumption of marine foods and their long-chain ω-3 fatty acids, as well as plant-based foods rich in bioactive compounds, as grape polyphenols, are involved in health promotion and disease risk reduction. In fact, fish oils and polyphenolic compounds are commonly used as supplements for both preventive and palliative strategies [2,4].

Marine ω-3 polyunsaturated fatty acids (PUFAs), mainly eicosapentaenoic acid (EPA; 20:5 ω-3) and docosahexaenoic acid (DHA; 22:6 ω-3), have shown protective effects against metabolic alterations induced by diet, including dyslipidemias, insulin resistance [13], and obesity [14]. They also exhibit anti-inflammatory properties, because they compete with ω-6 PUFAs leading to a displacement from the ω-6 to the ω-3 metabolic pathways [15] and can prevent oxidative damage of biomolecules, including proteins [16] and DNA [17], by enhancing the antioxidant system. 

Polyphenols from grape seed and other plants are bioactive compounds well-known mainly because of their antioxidant and anti-inflammatory properties [18] but also their effects on intestinal glucosidases and gut microbiota [19,20], which made polyphenols adequate for prevention of diet-related diseases.

Recent information collected from both in vitro and in vivo experiments indicate the additive effects of ω-3 PUFAs and grape polyphenols. Thereby, grape seed polyphenols extract (GSE) decreases fish lipids oxidation during the digestion and increases their intestinal bioavailability [21]. Moreover, the combination of ω-3 PUFAs and GSE regulate microbiota in healthy [19] and unhealthy [20] rats, reducing plasma insulin, leptin, and triglycerides levels, prevent perigonadal fat accumulation [22]. Interestingly, the combination also decreased inflammation by modulating eicosanoids and docosanoids production [15]. Additive effects between fish oils and polyphenols have been also found in the up- or down-regulation of the concentration of several liver proteins, especially when are added to a diet high in fat and sucrose [23]. In all those previous works, it was also demonstrated that the bioactivity of both ω-3 PUFAs and GSE is strongly influenced by the background diet, being either standard or high in fat and sucrose.

Considering that: (a) Protein carbonylation and obese-derived metabolic alterations are closely related; (b) fish oils [24] and GSE [25] have separately exerted an effect in modulating protein carbonylation; and (c) the additive effects of fish oils and GSE in several aspects of metabolism already study in the same cohort of rats, such as gut microbiota, regulation of protein expression and lipid mediator profiles [15,20,23]; the present study aimed to explore the effects of ω-3 EPA and DHA from fish oil mixture (FOM), GSE and their combination (FOM&GSE) on the liver carbonylome of rats given an obesogenic diet, i.e., high-fat and high-sucrose diet (HFHS), and a standard (STD) diet as background diets. The inclusion of STD diets in the experimental design allows us to test if the effect of supplements in modulating protein carbonylation is dependent on the background diet. This information will be useful for the correct design of both preventive and palliative nutritional strategies against metabolic disease induced by diet.

## 2. Results

### 2.1. Biochemical and Biometrics

#### 2.1.1. Effects of Fish Oil Mixture (FOM) and Grape Seed Polyphenols Extract (GSE) Supplements on HFHS-Fed Rats

A summary of the most relevant biochemical and biometric parameters of rats fed HFHS diets is shown in Table 1. These parameters have already been studied in the same cohort of animals and partially published [15,22,23]. Rats fed the HFHS control diet developed a prediabetic state characterized by hyperinsulinemia and excess of visceral fat [13,19]. Results indicated that none of the supplementations influenced weight increase as well as adiposity and hepatosomatic indexes. However, FOM significantly altered some cardiovascular (CDV) risk factors in plasma because reduced total cholesterol level and the amount of free fatty acids (FFA). Moreover, the FOM supplement significantly reduced plasma insulin concentration when was added in combination with GSE as compared to HFHS-C and HFHS-GSE groups. Regarding oxidative stress parameters in plasma, the combination of FOM&GSE improved the Oxygen Radical Absorbance Capacity (ORAC) and all supplementations significantly increased glutathione peroxidase (GPX) enzyme activity. Interestingly, the GPX activity drastically increased in the FOM&GSE group. However, FOM supplement increased the level of MDA in the liver. The combination of FOM with GSE significantly decreased that level, but remaining higher than in control and GSE supplemented diet. Finally, the three supplements improved liver inflammation parameters. In particular, the inclusion of fish oils triggered significantly lower concentration of TNFα. 

#### 2.1.2. Effects of Fish Oil Mixture (FOM) and Grape Seed Polyphenols Extract (GSE) Supplements on STD-Fed Rats

A summary of the most relevant biochemical and biometric parameters of rats fed STD diets is shown in Table 2. These parameters have already been studied in the same cohort of animals and partially published [15,19,23]. The supplementation of STD with FOM, GSE or both showed no changes in weight increase but rendered more adiposity index, especially with GSE supplementation. Regarding the plasma lipid profile, only the combination FOM and GSE significant decreased FFA levels, as compared to STD-control. Total cholesterol, triglycerides, TFA, and plasma insulin were unaltered. More differences were found regarding oxidative and inflammation parameters. The three supplements significantly increased plasma GPX enzyme activity, especially FOM&GSE, as previously mentioned for HFHS diets. FOM increased the level of MDA in the liver of STD-fed rats, and as in the case of HFHS diets, the inclusion of GSE counteracted that increment. Interestingly, FOM, GSE, and FOM&GSE significantly diminished liver inflammation, measured through CRP and TNFα, as compared to STD-C.

### 2.2. Modulation of Total Level of Carbonylation in Plasma by Fish Oil and Grape Polyphenol Supplementations

**Figure 1:**
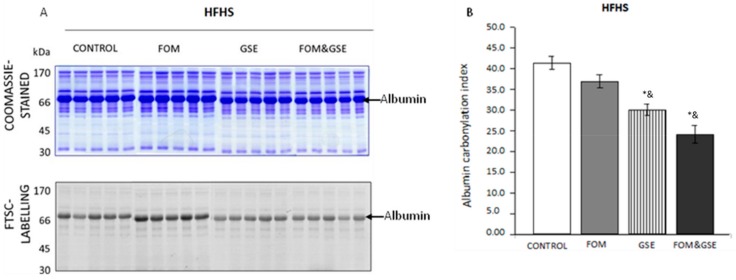
Effect of FOM, GSE, and FOM&GSE on albumin carbonylation index in plasma from rats fed HFHS diets. (**A**) 1-DE Coomassie-stained gel (upper panel A) and 1-DE FTSC-stained gel (lower panel A) of plasma proteins HFHS dietary groups. (**B**) Albumin protein carbonylation index measured in HFHS-CONTROL, -FOM, -GSE, and -FOM&GSE fed rats. Results are means (standard deviation) (*n* = 5–7). * *P* < 0.05 vs. HFHS-C; ^&^ *P* < 0.05 vs. HFHS-FOM; ^#^ *P* < 0.05 vs. HFHS-GSE; ^$^ *P* < 0.05 vs. HFHS-FOM&GSE. Comparisons were performed using the one-way ANOVA and Tukey post hoc tests. Images are representatives of three independent labelling experiments performed in triplicates. HFHS: high-fat and high-sucrose diet; GSE: grape seed polyphenols extract; FOM: fish oil mixture.

Total protein carbonylation in plasma of rats fed HFHS diets are shown in Figure 1. Albumin, which is the main protein in plasma, had also the strongest carbonylation signal (Figure 1A). Moreover, the level of albumin carbonylation was significantly modulated by supplements in the obesogenic diet. As Figure 1B shows, diet supplementation with GSE, itself or combined with FOM, significantly (*P* < 0.05) decreased albumin carbonylation as compared to HFHS-C and FOM groups.

**Figure 2:**
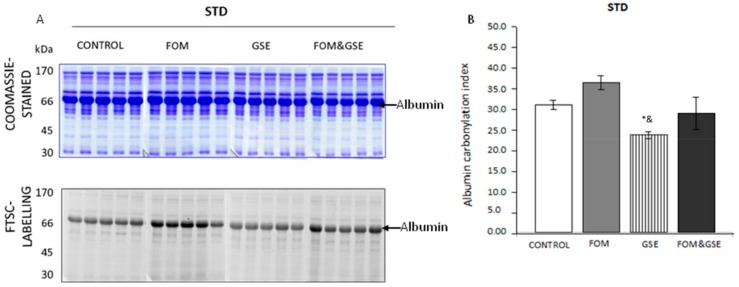
Effect of FOM, GSE, and FOM&GSE on albumin carbonylation index in plasma from rats fed STD diets. (**A**) 1-DE Coomassie-stained gel (upper panel A) and 1-DE FTSC-stained gel (lower panel A) of plasma proteins STD dietary groups. (**B**) Albumin protein carbonylation index measured in STD-CONTROL, -FOM, -GSE, and -FOM&GSE fed rats. Results are means (standard deviation) ((*n* = 5–7). * *P* < 0.05 vs. STD-C; ^&^ *P* < 0.05 vs. STD-FOM; ^#^ *P* < 0.05 vs. STD-GSE; ^$^ *P* < 0.05 vs. STD-FOM&GSE. Comparisons were performed using the one-way ANOVA and Tukey post hoc tests. Images are representatives of three independent labelling experiments performed in triplicates. STD: standard diet; GSE: grape seed polyphenols extract; FOM: fish oil mixture.

As for rats fed STD diets, albumin was also the main carbonylated protein in plasma and the one modulated by diet supplements, as it is shown in Figure 2. The effect of GSE for protecting albumin from carbonylation was detected in STD diets, being the carbonylation index of albumin significantly lower (*P* < 0.05) in rats supplemented with GSE (Figure 2B).

### 2.3. Modulation of Total Level of Carbonylation in Liver by Fish Oil and Grape Polyphenol Supplementations

**Figure 3:**
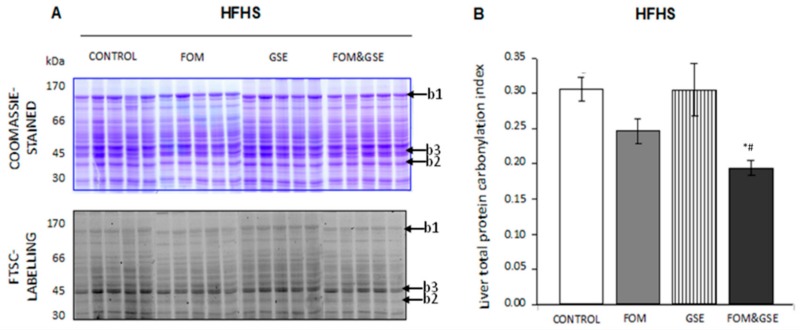
Effect of FOM, GSE, and FOM&GSE on protein carbonylation index in liver from rats fed HFHS diets. (**A**) 1-DE Coomassie-stained gel (upper panel A) and 1-DE FTSC-stained gel (lower panel A) of liver proteins HFHS dietary groups. (**B**) Liver total protein carbonylation index measured in HFHS-CONTROL, -FOM-, -GSE-, and -FOM&GSE-fed rats. Results are means (standard deviation) (*n* = 5–7). * *P* < 0.05 vs. HFHS-C; ^&^ *P* < 0.05 vs. HFHS-FOM; ^#^ *P* < 0.05 vs. HFHS-GSE; ^$^ *P* < 0.05 vs. HFHS-FOM&GSE. Comparisons were performed using the one-way ANOVA and Tukey post hoc tests. Images are representatives of three independent labelling experiments performed in triplicates. HFHS: high-fat and high-sucrose diet; GSE: grape seed polyphenols extract; FOM: fish oil mixture.

Total protein carbonylation was also determined in liver. Figure 3A corresponds to the SDS-PAGE images of liver proteins extracted from rats fed HFHS diets. Carbonylation of liver proteins was distributed from high to low molecular weights, being largely independent of protein concentration, as observed when superposing Coomassie-stained and FTSC-labelled gels. Some protein bands (such as b1 and b2) were highly concentrated but slightly oxidized, while some others (b3, for example) were highly carbonylated but scarcely concentrated, which indicates that protein carbonylation in quite independent of protein concentration, at least for some proteins.

Total protein carbonylation index values calculated for liver proteins in HFHS-fed rats are represented in Figure 3B. The supplementation of HFHS diet with FOM combined with GSE, significantly decreased (*P* < 0.05) carbonylation level for the sum of liver proteins, as compared to HFHS-C and the group supplemented just with GSE.

**Figure 4:**
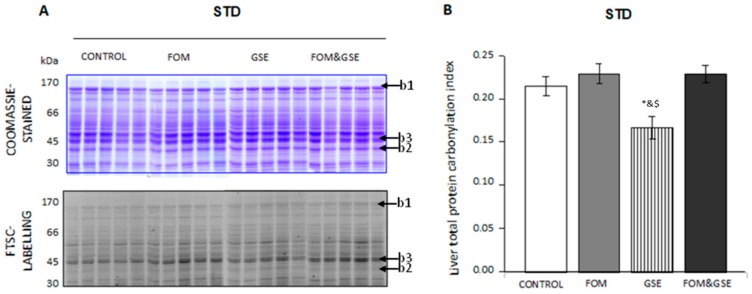
Effect of FOM, GSE, and FOM&GSE on protein carbonylation index in liver from rats fed STD diets. (**A**) 1-DE Coomassie-stained gel (upper panel A) and 1-DE FTSC-stained gel (lower panel A) of liver proteins STD dietary groups. (**B**) Liver total protein carbonylation index measured in STD-CONTROL, -FOM-, -GSE-, and -FOM&GSE-fed rats. Results are means (standard deviation) (*n* = 5–7). * *P* < 0.05 vs. STD-C; ^&^ *P* < 0.05 vs. STD-FOM; ^#^ *P* < 0.05 vs. STD-GSE; ^$^ *P* < 0.05 vs. STD-FOM&GSE. Comparisons were performed using the one-way ANOVA and Tukey post hoc tests. Images are representatives of three independent labelling experiments performed in triplicates. STD: standard diet; GSE: grape seed polyphenols extract; FOM: fish oil mixture.

In STD diets, FOM, GSE, and FOM&GSE-protein carbonylation profiles were similar to the STD-control, ranging from high to low molecular weights. Some high abundant proteins were poorly oxidized (bands 1 and 2, for instance) and vice versa (such as band 3), as it is shown in Figure 4A. Only the supplementation with GSE significantly decreased total liver carbonylation (Figure 4B). Thus, FOM seemed to be more effective in decreasing protein carbonylation when added to HFHS diets. On the contrary, GSE showed more efficiency in STD diets.

### 2.4. Focusing on the Protein Liver Carbonylome: Modulation of Specific Protein Carbonylation

The resolution of liver FTSC-tagged proteins on 2-DE gels allowed the identification of carbonylated proteins and the relative quantification of their specific carbonylation level.

**Figure 5:**
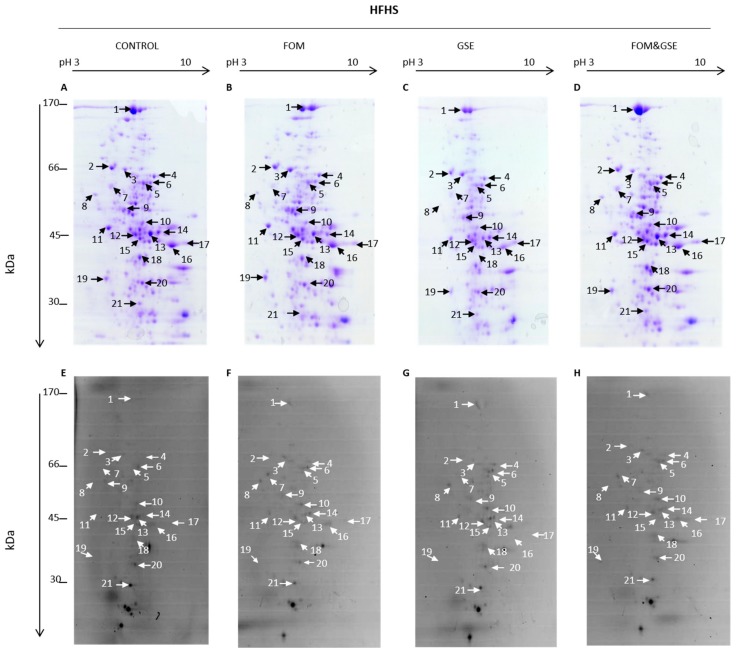
Representative 2-DE gels showing total and carbonylated protein profiles of liver detected in HFHS diets. Coomassie-stained 2-DE gels from (**A**) HFHS-CONTROL, (**B**) -FOM, (**C**) -GSE, and (**D**) -FOM&GSE. FTSC-stained 2-DE gels from (**E**) HFHS-CONTROL, (**F**) -FOM, (**G**) -GSE, and (**H**) -FOM&GSE. Numbered protein spots represent carbonylated proteins confidently identified, and they are listed in Table 3. Images are representatives of three independent labelling experiments performed in triplicates. HFHS: high-fat and high-sucrose diet; GSE: grape seed polyphenols extract; FOM: fish oil mixture.

Liver protein carbonylome of HFHS-fed rats is shown in Figure 5, where panels A, B, C, and D represent total proteins (Coomassie-stained gels) and panels E, F, G and H represent those carbonylated proteins from the total set of proteins (FTSC-labelled proteins) found in control, FOM, GSE, and the combination FOM&GSE HFHS-fed rats, respectively.

**Figure 6:**
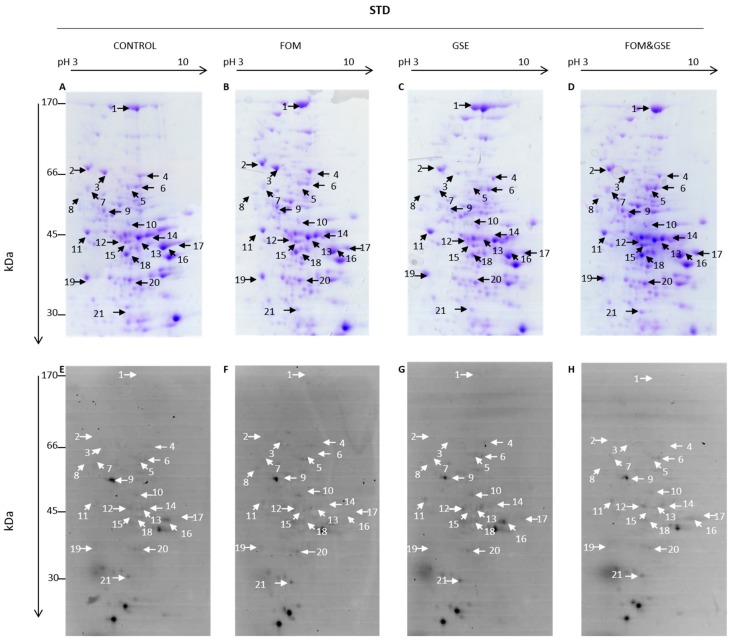
Representative 2-DE gels showing total and carbonylated protein profiles of liver detected in STD diets. Coomassie-stained 2-DE gels from (**A**) STD-CONTROL, (**B**) -FOM, (**C**) -GSE, and (**D**) -FOM&GSE. FTSC-stained 2-DE gels from (**E**) STD-CONTROL, (**F**) -FOM, (**G**) -GSE, and (**H**) -FOM&GSE. Numbered protein spots represent carbonylated proteins confidently identified, and they are listed in Table 3. Images are representatives of three independent experiments performed in triplicates. STD: standard diet; GSE: grape seed polyphenols extract; FOM: fish oil mixture.

Likewise, liver protein carbonylome from STD-fed rats is shown in Figure 6. Panels A, B, C, and D correspond to Coomassie-stained gel images, while panels E, F, G and H correspond to FSTC-labelled proteins from control, FOM, GSE, and FOM&GSE STD-fed rats.

The same proteins were identified as targets of carbonylation in both HFHS- and STD-fed rats and represented about the ~10% of the total number of proteins resolved on 2-DE gels. Protein identifications are shown in Table 3, where Spot *N*° refers to spot numbers indicated by arrows in Figure 5 and Figure 6. Carbonylation index calculated for each protein and supplement in HFHS and STD diets are reported in Table 4 and Table 5 respectively.

Carbonylation indexes calculated for each protein in the HFHS subgroups are shown in Table 4. In rats fed HFHS diets, 12 spots significantly changed (*P* < 0.05) their carbonylation index among the four different subgroups. However, each supplement exerted different modulation depending on the specific protein. As compared to control, FOM supplementation significantly reduced carbonylation of serum albumin (Alb) (spot 3), mitochondrial aldehyde dehydrogenase (Aldh2) (spot 9), the proteins contained in spot 12, i.e., mitochondrial long-chain specific Acyl-CoA dehydrogenase (Acadl), 4-hydroxyphenylpyruvate dioxygenase (Hpd) and beta-ureidopropionase (Upb1), and mitochondrial ornithine carbamoyltransferase (Otc) (spot 18). GSE supplementation reduced carbonylation of serotransferrin (*T*f) (spot 2), catalase (Cat) (spots 5,6), and the mixture of proteins detected in spot 12 (Acadl, Hpd, and Upb1). Besides those effects for decreasing protein carbonylation, FOM and GSE elevated the carbonylation level of some liver proteins, as 6-phosphogluconate dehydrogenase decarboxylating (Pgd) (spot 10), cytoplasmic actin 1(Actb) (spot 11), and omega-amidase NIT2 (Nit2) (spot 21), for FOM, and Aldh2, mitochondrial aspartate aminotransferase (Got2) (spot 17), and 3-alpha-hydroxystaroid dehydrogenase (Akr1c9) (spot 20), for GSE.

The detailed analysis of the effects of the combination FOM and GSE on the modulation of protein carbonylome, revealed some important information about the existence of potentially synergistic effects between both supplements. Considering the proteins whose carbonylation indexes changed by the combination of FOM and GSE, we can establish three different subsets of proteins:

(a) Proteins which were mainly sensitive to the oxidative changes induced by one but not both supplements. Proteins belonging to this first subset were albumin, actin, Otc, and catalase, because FOM supplementation explained the behavior of the three first and GSE seemed to be the main responsible for the decrease of catalase carbonylation.
(b) Proteins whose changes in carbonylation index can be explained by the “direct sum” of individual effect of the supplements (additive effect). We can include in this subset: Aldh2, because FOM and GSE showed opposite individual behavior and the combination did not show any effect compared to HFHS control; the mixture of proteins corresponding to spot 12, because both supplements exerted the same individual effects that were exacerbated after their combination. Got2 and Akr1c9 could be also included in this group. GSE increased their carbonylation index but the addition of FOM, which did not show any individual effect, made the proteins reached the carbonylation level of controls. Therefore, the addition of FOM seemed to counteract the GSE effect on these proteins.
(c) Proteins that showed an “unexpected” response to the combination of supplements (synergistic effect). Serotransferrin was one of the proteins belonging to this group because the combination FOM&GSE increased its carbonylation, while individual supplementation with FOM did not have any response and GSE significantly decreased its carbonylation level. The second protein in this group was the mitochondrial 60 kDa heat shock protein (Hspd1) (spot 7), which significantly reduced its carbonylation in the FOM&GSE group but individual FOM and GSE supplements did not exert any effect. Finally, Pgd and Nit2 significantly decreased its carbonylation level in the FOM&GSE-HFHS diet, but FOM supplement exerted the opposite effect increasing protein carbonylation and GSE did not show any measurable effect.

Carbonylation index calculated for each protein in the STD subgroups are shown in Table 5. With regards to the effects on the STD diets, 17 spots significantly changed (*P* < 0.05) their carbonylation indexes by the inclusion of one of the supplements. As compared to STD-Control, FOM significantly reduced the carbonylation index of Alb and Got2. GSE supplementation decreased carbonylation level of Hspd1, Pgd, Akr1c9, and Otc. FOM and GSE supplements increased the carbonylation of some proteins as well. FOM elevated the carbonylation of protein disulfide-isomerase (P4hb) (spot 8) while GSE increased Actb, catalase and Got2 carbonylation. 

Regarding the group that received the combination of FOM and GSE, carbonylated proteins can be also classified into three subsets of proteins, following the same criteria as described above for HFHS diets:

(a) Proteins which were mainly sensitive to one but not both supplements: Actb, which carbonylation index increased only through the GSE supplementation, and Nit2, whose carbonylation was reduced by FOM supplements.
(b) Proteins whose changes can be explained by the sum of the individual effects of supplements (additive effect). This group included P4hb, Akr1c9, albumin, Hsp1, transketolase (Tkt) (spot 4), Pgd, alcohol dehydrogenase 1 (Adh1) (spot 16), and catalase, which were significantly influenced by one supplement but not the combination.
(c) Proteins that showed an “unexpected” response to the combination of supplements (synergistic effect). The proteins included in this group were: Aldh2 and argininosuccinate synthase (Ass1) (spot 13), which presented higher carbonylation index after the combination of FOM and GSE; Got2 and fumarylacetoacetase (Fah) (spot 15), which reached the highest carbonylation level in FOM&GSE group even if FOM reduced its carbonylation and GSE increased it. Additionally, the mixture of proteins identified in the spot 12 (Acadl, Hpd, and Upb1) showed the lowest carbonylation value with the combination of both supplements and finally, Otc, which significantly increased its carbonylation whereas GSE produced the opposite effect and FOM did not affect.

### 2.5. Protein–Protein Interaction (PPI) Network of Carbonylated Liver Proteins and Functional Enrichment Analysis

**Figure 7:**
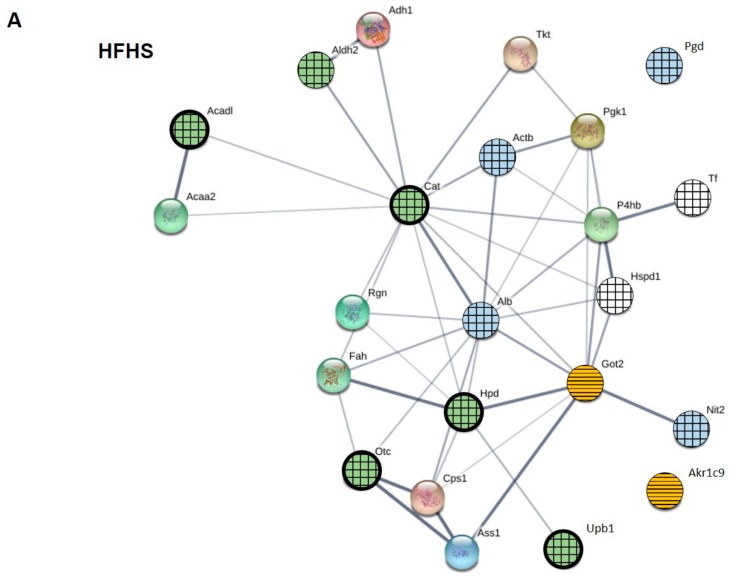

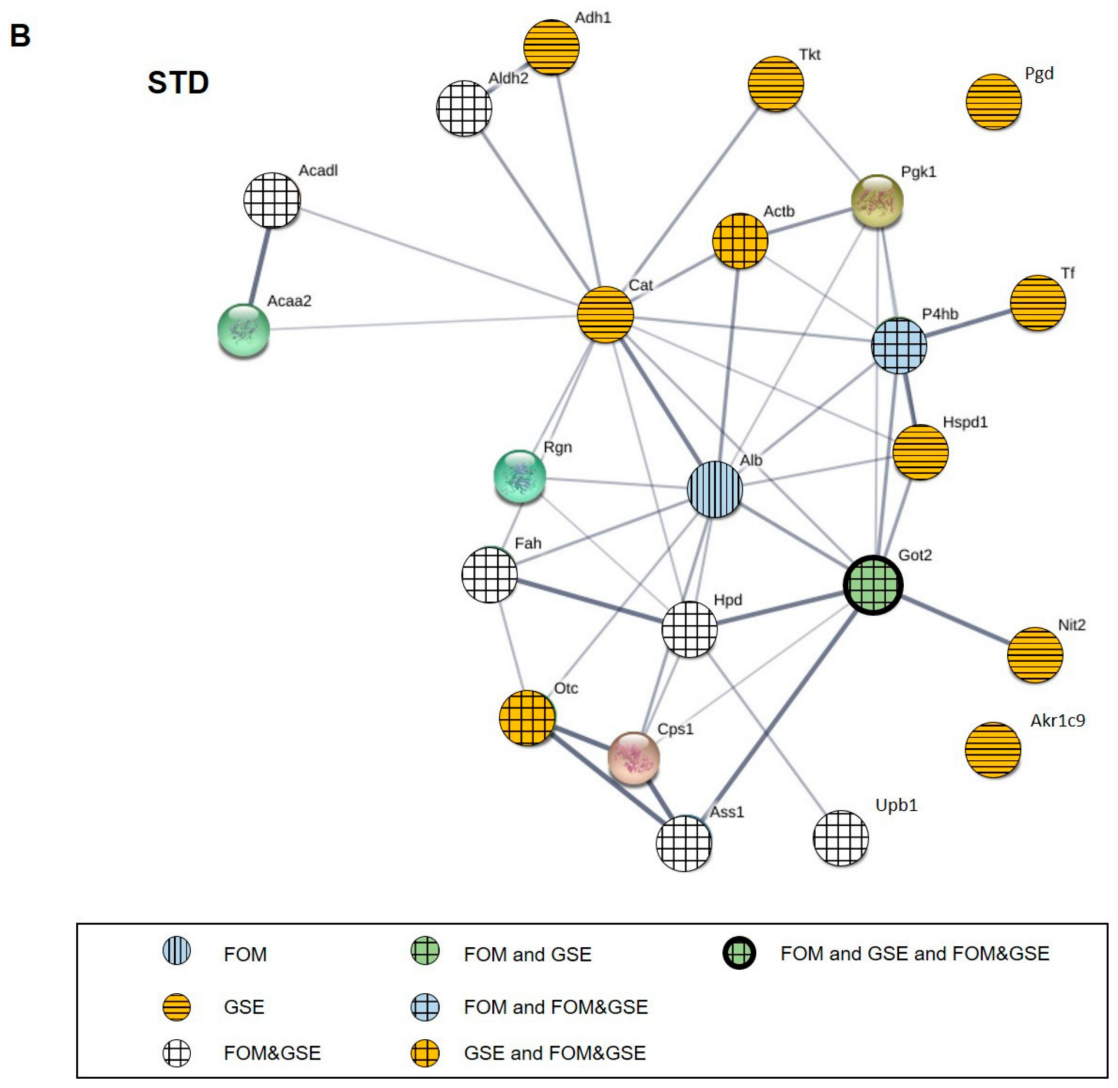
Protein–protein interaction network of liver carbonylated proteins obtained by using the STRING software. (**A**) Proteins whose carbonylation level was modulated by fish oil mixture (FOM), grape seed polyphenols extract (GSE) and both when they were added to high-fat and high-sucrose (HFHS)-diets. (**B**) Proteins whose carbonylation level was modulated by FOM, GSE and both when they were added to standard (STD)-diets. Nodes (circles) represent carbonylated proteins list in Table 3 and are labelled according to gene name. Lines (edges) indicate the known interrelationships. Thicker lines represent stronger associations.

The protein–protein interaction (PPI) network of the carbonylated proteins identified in rat liver (Table 3) was visualized by STRING (https://string-db.org/). The resulting network is represented in Figure 7. Proteins modulated by supplements in HFHS diets are highlighted in panel A while proteins modulated by supplements in STD diets are highlighted in panel B. Nodes (23) represent the carbonylated proteins and edges (47) correspond to known functional associations based on information contained in the STRING database. The STRING database includes experimental data, computational prediction methods and public text collections. Moreover, the thickness of the edges represents the confidence score of each functional association. 

The network of liver carbonylated proteins had significantly more interactions (47) than expected (5), resulting in a PPI enrichment *p*-value < 1.0 × 10^−16^. This significant enrichment indicates that liver carbonylated proteins have more interactions among themselves than what would be expected for a random set of proteins of similar size, drawn from the genome [26]. In other words, such enrichment means that liver carbonylated proteins are biologically connected, at least in part, as a group, as it is described in the functional enrichment analysis reported in the Supplementary Table S1. The topological analysis of the network in Figure 7 reflects also this situation. Only the proteins Pgd and Ak1rc9 did not show any functional association while the rest of carbonylated proteins are highly interconnected. Albumin and catalase, with 11 direct functional associations each, Got2, with 9 edges, and P4hd, with 8, occupy a central position in the network interconnecting all rest of carbonylated proteins. Moreover, highlighting with different colors the proteins differentially modulated by supplements (blue for fish oil and yellow for GSE) make easier to notice that fish oils seemed to have a more relevant action in HFHS and GSE seemed more active in STD. Results from total protein carbonylation showed in Section 2.3 led to the same conclusion.

It has been reported that the functions and activities of a growing number of proteins can be altered by their oxidation state, and some types of carbonylation, as lipoxidation, are starting to be considered as a mechanism of protein regulation [27]. Carbonylation of a certain protein usually leads to loss of function. Hence, changes in its carbonylation level will affect its function but also may compromise some pathways and processes in which the protein participates, especially if the carbonylation level of a significant number of other proteins from the same pathway or process changed as well. Functional enrichment analysis can also help to understand which proteins are more prone to carbonylation and design strategies of analysis more addressed. Therefore, functional enrichments in terms of pathways and biological processes were investigated in the subgroup of carbonylated proteins affected by each supplement in HFHS and STD diets by using Metascape (http://metascape.org/), according to the parameters described in Material and Methods section. 

In HFHS diets, 50% of proteins altered by FOM have oxidoreductase activity, 10% are transferases and the rest are hydrolases, transfer/carrier proteins and cytoskeletal proteins, and globally participate in small molecule catabolic process, drug catabolic process, response to nutrient, monocarboxylic acid metabolic process and innate immune response. In the case of GSE, 50% of proteins that respond to supplementation were also oxidoreductases, 10% hydrolases, 10% transferases and the rest were receptor/transfer/carrier proteins. This subgroup of proteins was mainly involved in processes related to the catabolism of small molecules, metabolism of alcohol, protein homooligomerization, metabolism of ketones and in the development of the liver and the response to insulin. Finally, in the case of the combination of FOM&GSE, oxidoreductases represented 33% of changed proteins, hydrolases, transferases and transfer/carrier protein the 17% each and the rest were a receptor and a cytoskeletal protein. These proteins principally participate in cellular amino acid processes, the catabolism of small molecules, respond to nutrients, the vesicle-mediated transport, metabolism of monocarboxylic acids and the innate immune system.

In STD diets, FOM only modulated the carbonylation of Alb, a transfer/carrier protein, and P4hb, involved in protein folding and the response to endoplasmic reticulum stress, but none pathways and processes resulted enriched. As for GSE, the 46% of affected proteins were oxidoreductases, the 18% transferases and the rest, a lyase and a cytoskeletal protein; being proteins principally involved in the metabolism of carbon, and the metabolic processes of cellular aldehydes and the glutamine family amino acid. Also, there are proteins that respond to toxic substances and insulin and participate in the innate immune system. The combination of FOM and GSE in STD diets also preferentially modified oxidoreductases (50%) and the rest were transferases, ligases, and cytoskeletal proteins; this subgroup of proteins was primordially involved the metabolism of alpha-amino acids, the arginine biosynthesis and in the response to toxic substances and fatty acids. 

A detailed list of pathways and processes enriched in the subset of carbonylated proteins modulated by each supplement is provided in the Supplementary Table S1.

## 3. Discussion

The present research was performed to pinpoint the modulation of protein carbonylome in the liver exerted by ω-3 PUFAs from FOM, GSE and their combination considering an obesogenic diet, i.e., high-fat and high-sucrose diet, and a standard diet as background diets. Our experimental design allowed us to explore how FOM plus GSE affect protein carbonylation in the liver and determine if the background diet plays a role in the bioactivity of those supplements concerning the carbonylome regulation. In previous studies performed with the same cohort of rats, we already demonstrated that the background diet influenced the function of ω-3 PUFAs and polyphenols in the up- and down-regulation of the expression of several liver proteins [23] and also in the regulation of lipid profiles in plasma, tissues and membranes, as well as eicosanoids and docosanoids synthesis [15]. 

The study described here addressed both protein carbonylation in general in the liver and carbonylation at specific protein as a function of the redox status induced by supplements. Considering firstly total protein carbonylation, we found that the inclusion of marine ω-3 PUFAs in the HFHS diet seemed the main responsible for decreasing carbonylation index, being especially effective in the FOM&GSE group. That effect was accompanied by the diminution of biomarkers of inflammation (TNFα and CRP) and the improvement of plasma lipid profile (lesser level of total cholesterol and FFA). Additionally, GSE could exert a higher influence on plasma since the significant decline of plasma albumin carbonylation induced by GSE was also detected in the combined diet. Moreover, FOM but especially GSE and their combination FOM&GSE significantly improved other oxidative parameters in plasma, including ORAC and GPX. Noteworthy, the combination of ω-3 PUFAs and GSE drastically increased plasma GPX activity, supporting a potential synergistic effect on this enzyme activity, as we previously described [15]. That increase in the activity of GPX can be explained because both FOM and GSE individually activate this enzyme; EPA and DHA likely via the production of hydroperoxides, and GSE via the production of hydrogen peroxide [28] but also up-regulating the nuclear factor-erythroid 2-related factor 2(Nrf2) [29].

Regardless total carbonylation measured in liver, identification and quantification of specific carbonylated proteins reflected that (a) the combined (additive, inhibitory or synergistic) action between supplements existed also on determinant proteins and is protein-depended (as we have described in detail in the result section); and (b) some proteins resulted less carbonylated but some others more in response to the same supplement in both HFHS and STD diets. This selectivity was previously reported in the liver of rats suffering from diet-related metabolic disorders [11,30].

The explanation behind the combined effect of supplements and their selective behavior on liver proteins can be found if considering how the carbonylation process occurs. Oxidation patterns in protein carbonylation are highly protein specific in terms of the dominant (and others) oxidation mechanism responsible for the induction of this post-translational modification (direct oxidation, reaction with lipid peroxidation products, AGEs), the amino acid which is being carbonylated and the carbonylation motif in each process [7]. Subcellular localization, molecular function of the protein, protein expression and degradation rates, or protein conformation are other critical factors to taking into account. Madian et al. [31] even demonstrated that the extent of oxidation at any one residue as a function of increasing oxidative stress is quantitatively independent of that at other residues in the same protein, existing then some sites drastically more labile than others. They also reported the lack of relationship between the mole fraction of carbonylation across all sites in a protein and changes at individual residues.

Since the consumption of fish oil induced the accumulation of ω-3 PUFAs in the liver while significantly reduced the amounts of ω-6 PUFAs as compared to control and GSE groups [15], the profiles of reactive carbonyls coming from lipid peroxidation likely changed in the FOM groups, and then, protein carbonylation profiles as well. 4-hydroxy-2-nonenal (4-HNE), a major cytotoxic product of lipid peroxidation, derivates from ω-6 fatty acid and readily forms covalent modifications of numerous targets [4]. Potentially, groups fed fish oil would generate less 4-HNE and the carbonylation of the proteins more labile to this electrophile aldehyde decreased. At least the enzyme Aldh2, involved in 4-HNE detoxification and which was less carbonylated in FOM group, is known to be modified by 4-HNE itself [32]. On the other side, the inclusion of fish oil elevated MDA levels. Although MDA is also highly reactive [27], the carbonylation of most of the proteins in FOM group did not increase and only actin and Pgd resulted in more carbonylated than control. Actin has a highly reactive Cys374 residue through which scavenges reactive electrophilic aldehydes through its without undergoing significant polymerization impairment [33]. Its elevated carbonylation in FOM groups can be maybe the consequence of acting as a protein carbonyl scavenger of MDA, preventing its covalent adduction with more vulnerable cytoplasmic proteins, which would lead to damage. The no increased carbonylation in other cytoplasmic proteins plus the lesser carbonylation of regucalcin, another cytoplasmic protein, are in agreement with this observation. Moreover, the enhancement of antioxidant activities should contribute. Regarding Pgd, its subcellular localization into the mitochondria and maybe more individual susceptibility to MDA than other mitochondrial proteins might explain its increased carbonylation in FOM-HFHS group. In agreement, in GSE groups, MDA level tended to be lower in HFHS and significantly lower in STD; and Pgd carbonylation did not increase and significantly decreased, respectively. GSE seemed to be effective in detoxification this aldehyde, may be exerting a direct action (for example, by chelation) or by increasing glutathione peroxidase detoxification. In fact, GSE significantly increased GPX activity in plasma (Table 1) and resulted in a lower level of MDA. This antioxidant effect was potentiated by the combination with fish oil because FOM supplementation likely reduced 4-HNE formation, as discussed before. Accordingly, Pgd carbonylation significantly decreased in FOM&GSE-HFHS group, which also exhibited the lowest total carbonylation, the highest number of proteins less carbonylated and the best antioxidant status according to TBARS and ORAC values presented in Table 1. 

On the other side, our previous results carried in the same cohort of rats demonstrated a down-regulated expression in proteins from the proteasome in these FOM&GSE rats [23]. A selective declined degradation of some oxidized proteins, as it has previously described [34], could explain the increased carbonylation of serotransferrin found in these rats. 

Besides changes in reactive carbonyls profiles, subcellular localization of proteins is important in protein carbonylation because ROS can be formed preferentially in some cell compartments and have different stability, reactivity and diffusion capacity [35]. For example, in HFHS diets, GSE reduced the carbonylation of catalase, a peroxisomal protein, as compared to control. This effect may be explained because it has been found that grape polyphenols can inhibit, at least in vitro, the xanthine oxidase (XO) system into peroxisomes [36]. This inhibition was maintained in FOM&GSE group, where it was also an increase of catalase expression [23]. It should be noted that the effect of polyphenols on XO depends on many factors, from concentration to specific polyphenol compound [36]. The differences found between STD and HFHS regarding polyphenols bioavailability can explain why polyphenols increased catalase carbonylation in STD diets [20]. When the HFHS diet was supplemented with both FOM&GSE, less catalase carbonylation index was measured accompanied by an up-regulation of catalase levels [23]. In the differential protein expression analysis, we reported that FOM and the combined FOM&GSE supplement potentiated peroxisomal fatty acid beta-oxidation [23]. Minor oxidation of catalase found in the current study plus the up-regulation of enzyme levels detected in the quantitative proteomic study previously performed [23] may result in a highly effective peroxisomal H_2_O_2_ detoxification for the FOM&GSE group. 

We hypothesize that the increased carbonylation in some proteins induced by GSE in HFHS diets can be a consequence of the higher carbonylation of Aldh2 and Akr1C9, both enzymes involved in the detoxification of 4-HNE [37], whose activity can be compromised by carbonylation induced by 4-HNE itself, exacerbating the problem. At least for Aldh2, it has been demonstrated that its activity is inhibited by toxic compounds such as 4-HNE [32]. This explanation can be also valid for the higher carbonylation of Got2. 

It has been described that the suppression of Got2 in hepatocytes can attenuate lipotoxicity induced by palmitate [38]. Therefore, the higher level of carbonylation of Got2 induced by GSE could protect the liver from the lipotoxicity induced by the high amount of palmitic acid in this diet [15], if the carbonylation damage compromises its activity.

In plasma, the major protein, which is albumin, reflected well the redox status described in HFHS diets. Albumin has been previously reported as a target of FOM in plasma and liver, decreasing carbonylation after supplementation of a diet with a mix of EPA and DHA [24]. GSE supplementation significantly reduced albumin carbonylation in plasma, but no significant effect was noticed in the liver. Therefore, FOM was more efficient in reducing albumin carbonylation in the liver and GSE was more efficient in plasma. Regarding FOM&GSE-HFHS, additive effects of individual supplements were clearly observed in the plasma, since albumin carbonylation index in those rats was the least among the four HFHS subgroups. The lesser albumin carbonylation in FOM&GSE-HFHS-fed rats reflected the significant improvement in their antioxidant status described in Section 2.1.2. but also contributed to that improvement because albumin is an important antioxidant protein [39].

In STD diets, the effects of FOM, GSE and FOM&GSE on carbonylome were different from the observed when they were added to HFHS. Consequently, it seems that the bioactivity of both FOM and GSE in modulating protein carbonylation are largely depended on the fat and sugar content of the dietary background. In previous studies performed with the same cohort of rats, we demonstrated that the background diet also influenced the way in the supplements modulated protein levels [23] and tissue lipidome [15]. Quantitative proteomics tools revealed that fish oils supplementation was the main force in regulating liver protein levels while GSE can modulate FOM activity [23]. Lipidomic analysis reflected the lesser susceptibility of fish oil bioactivity to be affected by the background diet and the greater susceptibility of GSE bioactivity [15]. The present results demonstrated that the background diet also affects another aspect of FOM and GSE bioactivity, i.e., modulation of carbonylome. These results evidence the importance of the omics approach to obtain the closest picture of the complex way in that bioactive compounds influence metabolism. GSE showed higher bioactivity in rats fed an STD than rats fed an HFHS diet. Consistently, we previously demonstrated that HFHS diets reduced the amounts of polyphenol metabolites bioavailable and bioaccessible in the gut and the amounts of many microbial metabolites of GSE were significantly increased in the STD–GSE group, as compared to HFHS-GSE group [20]. In consequence, besides the differences in the main pathways modulated by GSE, we observed that GSE increased catalase carbonylation level while decreasing aldo-keto reductase and Pgd in STD, just the opposite effect to the one measured into HFHS diets. Interestingly, our previous study showed scarce bioactivity of GSE in inducing changes of relative expression levels of liver proteins [23]. In consequence, our current data indicated that GSE action on proteome in STD is mainly due to the modulation of protein carbonylation status and maybe enzyme activities rather than expression. Noteworthy, FOM in combination with GSE drastically promoted GPX activity, revealing a synergistic action between both supplements on that enzyme, as described above for HFHS diets. Moreover, the FOM&GSE supplementation mitigated the lipid accumulation provoked by GSE in the STD background (see adiposity index and TFA measured in Table 2), while preserving the ability of FOM and GSE to prevent inflammation, demonstrating the benefits of combining the single healthy properties of fish oils and grape polyphenols also when STD is the background diet. 

## 4. Materials and Methods

### 4.1. Materials and Reagents

Fish oil mixture (FOM) was obtained by mixing adequate quantities of commercial fish oils AFAMPES 121 EPA (AFAMSA, Vigo, Spain), EnerZona Omega 3 RX (ENERZONA, Milano, Italy) and Oligen liquid DHA 80% (IFIGEN-EQUIP 98, Barcelona, Spain) to obtain a final ratio of 1:1 EPA/DHA. The sum of EPA and DHA was 50% of total fatty acids. The fatty acid composition was confirmed then by gas-liquid chromatography and was the same than previously published [15,23]. Because PUFAs are easily oxidized and the oxidation by-products are potentially toxic, the peroxide value of the oils administered was checked periodically. It was below 5 mEq oxygen per kilogram of oil throughout the interventional study. The doses of fish oil were given according to the European Union’s recommendation on ω-3 PUFA.

GSE Grajfnol (≥95% proanthocyanidins, 85% oligomers) was from JF- Natural Product (Tianjin, China). The used dose of GSE was 30 mg proanthocyanidin/kg body weight of rat. This dose, extrapolated to humans, would be a daily dose of 4.9 mg/kg body weight, 340 mg/day for an adult weighing 70-kg adult [40], and the median daily polyphenol intake in humans is from about 150 to nearly 500 mg/polyphenols/day, as previously described [41].

Ketamine-HCl was purchased from Merial Laboratorios S.A. (Barcelona, Spain) and ProteoBlock^TM^ Protease Inhibitor Cocktail from Thermo Fisher Scientific Inc. (Rockford, IL, USA). Fluorescein-5-thiosemicarbazide (FTSC) was acquired from Invitrogen (Carlsbad, CA, USA), the sequencing-grade trypsin from Promega (Madison, WI, USA) and an internal standard of nonadecanoic acid (19:0) from Larodan Fine Chemicals (Malmö, Sweden). Acrylamide and bis-*N*,*N*-methylene-bis-acrylamide were purchased from Bio-Rad Laboratories (Hercules, CA, USA). Immobiline DryStrip gels (IPG strips) of pH range 3–10 and 11 cm, IPG buffer, pharmalyte 3–10, bromophenol blue and TEMED were obtained from GE Healthcare Bio-Sciences AB (Uppsala, Sweden). Bicinchoninic acid (BCA) and Bio-Rad protein assays, phenylmethylsulfonyl fluoride (PMSF), dithiothreitol (DTT), iodoacetamide (IA), ethylenediaminetetraacetic acid (EDTA), trichloroacetic acid (TCA), Tris Hydrochloride (Tris–HCl) and CHAPS detergent were obtained from Sigma (St. Louis, MO, USA).

### 4.2. Animals and Diets

Fifty-six 8-10-week-old Wistar Kyoto rats (Charles Rivers Laboratories, Wilmington, MA, USA) were housed in animal cages (*n* = 2–3/cage) with a constantly regulated temperature (22 ± 2 °C) and humidity (50% ± 10%) with a 12-h light–12-h dark cycle. The rats were arbitrarily assigned to either an STD group (*n* = 28), which fed a standard diet based on the reference diet Teklad Global 2014 (Harlan Teklad Inc., Indianapolis, IN, USA), or an HFHS group (*n* = 28), which were given an HFHS based on the TD 08,811 diet (Harlan Teklad Inc.). Both groups were also divided into four subgroups (*n* = 7) according to the added supplement: control (C) groups, which fed an STD or HFHS diet supplemented with soybean oil; FOM groups, which fed an STD or HFHS diet supplemented with FOM contained a ratio of 1:1 EPA/DHA; GSE groups, which fed an STD or HFHS diet supplemented with proanthocyanidin-rich GSE and finally; FOM and GSE groups (FOM&GSE), which fed an STD or HFHS diet supplemented with a combination of EPA/DHA EPA/DHA 1:1 and GSE.

During the 24-week-length experimental period, water and food were provided *ad libitum* and consumptions were daily registered. After fasting all-night, rats were anesthetized by intraperitoneal injection with 80 mg of ketamine per kg of body weight and 10 mg of xylazine per kg of body weight before killing by exsanguination. Blood was collected from the saphenous vein in EDTA containing tubes. Plasma was obtained after centrifugation and PMSF was added to prevent proteolysis, plasma samples were stored at −80 °C. Livers were excised, perfused with 0.9% NaCl solution, weighed and quickly frozen in liquid N_2_. Livers were also kept at −80 °C until the moment of analyses. 

All the procedures strictly followed the European Union guidelines for the care and management of laboratory animals, striving to minimize suffering, and were approved by the CSIC (Spanish Research Council) Subcommittee of Bioethical Issues (Ref. AGL2009-12374-C03-03).

### 4.3. Biochemical Measurements

Plasma total fatty acids (TFAs) and free fatty acids (FFAs) were analyzed as previously described [42,43]. Plasma triglycerides, total cholesterol, LDL and HDL joint to plasma insulin and glucose concentrations were measured in the same animals by following protocols previously described [44,45,46]. Total MDA was measured by HPLC- fluorescence [47] just after protein hydrolysis [48] and precipitation [49] prior to derivatize with thiobarbituric acid (TBA). Plasma antioxidant capacity was measured as the ORAC [50] and the activities of GPx in plasma were also assayed [51]. ELISA kits from Cusabio Biotech (Hubei, China) were used to measure CRP and TNFα in the liver. Data were reported as mean and standard deviation (SD). Statistical analysis was performed by using the one-way analysis of variance (ANOVA) followed by Tukey’s Post Hoc Test with the IBM SPSS Statistics 22 software (Statsoft, Tulsa, OK, USA). Comparisons among different supplements in the present research were made independently in STD or HFHS backgrounds.

### 4.4. Extraction of Liver Proteins and FTSC Labelling of In Vivo-Generated Protein Carbonyls in Both Liver and Plasma 

Proteins were extracted from 200 to 400 mg of liver in 25 volumes of a buffer contained 20 mM sodium phosphate, pH 6.0, 0.5 mM MgCl2, 1 mM EDTA, and 10 μL/mL of the ProteoBlock^TM^ Protease Inhibitor Cocktail (Thermo Fisher Scientific Inc., Rockford, IL, USA), which comprised a mixture of 100 mM AEBSF–HCl, 80 mM aprotinin, 5 mM bestatin, 1.5 mM E64, 2 mM leupeptin, and 1 mM pepstatin A. Sample extraction was made by using an Ultra-Turrax high-performance disperser. After homogenization, samples were centrifuged at 100,000× *g* for 1 h at 4 °C and the bicinchoninic acid assay (BCA) was used to determine protein concentration [52].

To label protein carbonyls, 1 mM of FTSC was added to liver and plasma proteins and the mixture was incubated for 2.5 h at 37 °C in the dark. FTSC-labelled proteins were then precipitated to remove the excess of FTSC with an equal volume of 20% TCA (*v*/*v*). After centrifugation at 16,000× *g* for 10 min at room temperature, FTSC-labelled proteins were resolubilized in 2-DE buffer (7 M urea, 2 M thiourea, 2% Chaps, 0.5% Pharmalyte 3–10, 0.5% IPG 3–10 buffer, and 0.4% DTT). Bradford assay was used for measuring sample protein concentration [53]. 

### 4.5. Relative Quantification of Total and Specific Protein Carbonylation

Total protein carbonylation was evaluated in plasma and liver by resolving 20 µg and 30 µg of each FTSC-labelled sample respectively in 1-D SDS-PAGE (10% T, 2.6% C) [54] and running the gels in a Mini-PROTEAN 3 cell (Bio-Rad, Hercules, CA). Linear range for albumin quantification was determined (Supplementary Figure S1). Specific protein carbonylation in the liver was measured by separating FTSC-labelled proteins in 2-DE gels [24]. Briefly, 400 µg of protein was loaded onto 11-cm IPG 3–10 dry strips on an Ettan IPGphor II isoelectric focusing system (GE Healthcare, Chicago, IL, USA) and protein focusing was performed by applying the voltage/time profiles suggested by the manufacturer (GE Healthcare, Chicago, IL, USA). After focusing, the strips were equilibrated while cysteine were sequentially reduced and alkylated. The second dimension consisted of running the samples on SDS-PAGE (10% T, 2.6% C) in an Ettan Daltsix electrophoresis system (GE Healthcare, Chicago, IL, USA).

After electrophoresis, gels were exposed to a UV transilluminator (520-nm band-pass filter, 520DF30 62 mm) (Molecular Imager Gel Doc XR System; Bio-Rad, Hercules, CA, USA) to visualize carbonylated proteins and recording FTSC-gel image. Finally, total proteins in gels were stained with the Coomassie dye PhastGel Blue R-350 (GE Healthcare, Chicago, IL, USA) and destained. All gels from the same experiment were manipulated in parallel and staining and destaining times were optimizing to minimize the changes in gel size and guarantee the correct superposition of gels. 

### 4.6. Image Analysis, Carbonylation Index Calculation, and Statistics

1-DE gels for total protein carbonylation determination were analyzed by using LabImage 1D (Kapelan Bio-Imaging Solutions, Halle, Germany) considering total lane pixel intensity. 2-DE gels for relative quantification of specific protein carbonylation were analyzed with the PDQuest software version 7.4 (Bio-Rad, Hercules, CA, USA). To normalize protein carbonylation level and avoid possible quantification errors generated during the experimental procedure, the carbonylation index was estimated as previously described [11]. The carbonylation index is the result of dividing the fluorescence intensity in the FTSC-stained gels by the corresponding intensity signal measured in the Coomassie-stained gel.

Specific and total carbonylation protein levels were reported as mean and standard deviation (SD). Statistical analyses were performed by analysis of variance (ANOVA) with IBM SPSS Statistics version 24 software (SPSS, Chicago, IL, USA). Normal distribution and homogeneity of variance were evaluated. The means were further compared by Tukey post hoc tests. The level of significant difference was set at *P* < 0.05.

### 4.7. In-Gel Digestion and Carbonylated Protein Identification by nanoLC–ESI–IT–MS/MS

Proteins comprising the liver carbonylome were identified from the 2-DE gels. Spots of interest were manual and directly excised from gel onto the UV transilluminator, to confirm the correct superposition of gels. After cutting, spots were sequentially washed alternating cycles of hydration with water and dehydration with 100% acetonitrile. The protein contained in each spot was then overnight digested at 37 °C with a 0.5 µM solution of sequencing-grade trypsin (Promega, Madison, WI, USA) in 50 mM NH_4_HCO_3_ buffer, pH 8. The resulting tryptic peptides were then desalted by using Millipore^®^ Ziptips C18 (MilliporeSigma, St. Louis, MI, USA), dried and finally resuspended in 1% formic acid. 

Peptides were subjected to nano-LC ESI-IT-MSMS analysis by using a Dionex UltiMate 3000 Series (ThermoFisher, Rockford, IL, USA) coupled to a dual-pressure linear ion trap mass spectrometer LTQ Velos Pro with electrospray ionization (ESI) (Thermo Fisher, Rockford, IL, USA). The loading pump provided 0.1% formic acid in water at 10 μL/min and the injection volume was 5 μL. The chromatographic separation of peptides was performed on an analytical C18 column (Acclaim PepMap RSLC C18, 2 µm, 100 Å, 75 µm i.d. × 15 cm) with a trap-column (µ-Precolumn holder, 5 mm, with 30 µm i.d.) (Thermo Scientific, San Jose, CA, USA). Flow rate delivered by the nano-pump was set at 300 nL/min, being 0.1% formic acid in water the mobile phase A, and 0.1% formic acid in acetonitrile the mobile phase B. Peptide separation was accomplished under a 60-min linear gradient elution from 5% to 40% B. 

Tandem mass spectrometry (MS/MS) was performed with the LTQ Velos Pro mass spectrometer operated in data-dependent acquisition (DDA) mode. MS1 survey scans were acquired in the mass range of mass/charge ratio (*m*/*z*) 400 to 1600 Da, with the top six most-intense precursor ions with ≥2 charge state subjected to MS/MS analysis. Precursors were selected and fragmented in the ion trap by collision-induced dissociation (CID) with 35% normalized collision energy and an isolation width of 2 Da. Former target ions were excluded for 30 s. For instrument control and data acquisition, it was used Xcalibur 2.0 and Tune 2.2 software (Thermo Fisher Scientific, Inc.). The acquired tandem mass spectra were searched against the Uniprot- Swiss-Prot *Rattus norvegicus* database using PEAKS DB (Bioinformatics Solutions Inc., Waterloo, ON, Canada). Search criteria were: oxidation of methionine and carbamidomethylation of cysteine as variable modifications, trypsin as the proteolytic enzyme, maximum 2 missed cleavages per peptide and a mass tolerance of ±1 Da for precursor and ±0.6 Da for product ion scans. False discovery rate (FDR) for identifications were accepted if <1%.

### 4.8. Protein–Protein Interaction (PPI) Network and Pathway and Process Enrichment Analysis

The PPI network analysis of the liver protein carbonylome was performed by submitting the list of carbonylated proteins, using gene IDs as identifiers, and selecting *Rattus norvegicus* as the organism to the STRING (Search Tool for the Retrieval of Interacting Genes) software version 11.0 (http://stringdb.org/) [26]. The PPI network was created only with the query proteins. The thickness of the edges which are connecting the nodes in Figure 7 representing the strength of data support, with a minimum confidence score of 0.4 (medium level). 

Metascape (www.metascape.org) [55] was used to discover the pathways and biological processes significantly modulated by the supplements. The gene list of carbonylated proteins significantly modulated by each supplement whether in HFHS or STD groups (Table 4 and Table 5) was first converted into their corresponding *Rattus norvegicus* Entrez gene IDs using the version of the database updated on 14 August 2019. Then, the enrichment analysis was sequentially carried out selecting GO Biological Processes, KEGG Pathway and Reactome Gene Sets, as ontology sources. All genes in the genome have been used as the enrichment background. The enriched pathways and processes showed in the Supplementary Table S1 were the clusters formed as the result of the collection and grouping of the terms (pathways and processes) with a *p*-value < 0.01, a minimum count (gen/protein) of 3, and an enrichment factor > 1.5. In this case, the enrichment factor is the ratio between the observed counts and the counts expected by chance. The most statistically significant term within a cluster is chosen to represent the cluster. More in detail, *p*-values were estimated based on the accumulative hypergeometric distribution [56], and *q*-values by using the Benjamini–Hochberg procedure (the FDR method [57]) to consider multiple testings [58]. Kappa scores [59] were employed during the hierarchical clustering on the enriched terms as the similarity metric, establishing that a cluster is considered when sub-trees exhibit a similarity of >0.3.

## 5. Conclusions

FOM and GSE modulated the liver protein carbonylome by selectively controlling protein carbonylation level in rats fed an obesogenic diet, such HFHS. Modulation of liver protein carbonylome occurred in rats fed STD as background diet as well. The identification and quantification of proteins which are targets of these bioactive compounds constitute a good starting point to fully understand the bioactivity of FOM and GSE concerning protein carbonylation. However, the dramatic specificity of the process will require in the next studies the development of methods that allow us the identification and quantification of the carbonylated residue and the type of modification formed on it. The influence of FOM and GSE on protein carbonylome was accompanied by significant improvements in other markers of oxidative stress and inflammation, as well as the health status. The combination of both FOM and GSE rendered especially pronounced health benefits according to phenotypic parameters, where additive and synergistic effects on carbonylation of specific liver proteins were detected. Moreover, FOM seemed to be a more relevant factor involved in the modulation of protein carbonylome in HFHS diets. When STD was the background diet, the phenotypic effects of the supplements were more moderate than in HFHS diets but resulted in the mitigation of some oxidative stress and inflammation parameters. Noteworthy, results showed more bioactivity of GSE in modulating the liver protein carbonylome in those STD diets. As a result, the combination of the benefits of FOM alongside those attributed to GSE in both dietary frameworks resulted in a general improvement of the metabolic status in rats fed FOM&GSE. Finally, the effect of FOM, GSE, and their combination on the liver protein carbonylome was highly depended on the background diet, which differently modulated multiple aspects besides protein carbonylation, including gut microbiota, lipid profiles (native lipidome and lipid mediators) and protein expression. The study of the influence of the background diet seems critical for the correct design of both preventive and palliative strategies against obesity-related diseases. 

## Figures and Tables

**Table 1:** Biometric measurements, biochemical and oxidative stress parameters measured in rats fed high-fat and high-sucrose (HFHS) diets. C: control; GSE: grape seed polyphenols extract; FOM: fish oil mixture.

	HFHS-C	HFHS-FOM	HFHS-GSE	HFHS-FOM&GSE
Weight Increase (%)	101.8 (16.9)	114.9 (20.3)	93.6 (14.4)	117.4 (20.6)
Adiposity index (%) ^×^	6.62 (3.95)	6.12 (2.62)	4.98 (1.31)	4.32 (1.54)
Hepatosomatic index (%) ^∆^	2.4 (0.1)	2.4 (0.1)	2.3 (0.1)	2.7 (0.4)
Total cholesterol (mmol/L)	3.5 (0.3)	2.7 (0.5) *^#^	3.7 (0.3)	2.8 (0.3) *^#^
Triglycerides (mmol/L)	1.3 (0.2)	1.6 (0.3)	2.2 (0.2) *^&$^	1.5 (0.3)
FFA (μg/mL)	180.7 (24.2)	140.2 (15.9)	187.8 (4.5) ^&$^	136.6 (25.2) *^#^
TFA (μg/mL)	2442.2 (245.0)	2233.0 (425.4)	3764.9 (374.1) *^&$^	2101.4 (231.1)
HbA1c (%)	3.25 (0.14)	3.14 (0.12)	3.11 (0.12)	2.70 (0.70)
Fasting Glucose (mg/dL)	65.86 (3.63)	64.14 (6.87)	65.43 (4.69)	67.00 (3.92)
Plasma insulin (ng/mL)	2.0 (0.6)	1.6 (0.4)	2.6 (1.2)	1.2 (0.5) *^#^
ORAC (µmol Trolox/mL plasma)	17.7 (7.9)	10.7 (5.7)	19.3 (5.0)	24.0 (3.5) ^&^
Plasma GPX(U/g Hb)	0.4 (0.04)	3.8 (0.9) *^#$^	13.4 (1.9) *^&$^	36.7 (8.5) *^&#^
TBARS (mg MDA/kg liver)	3.6 (0.7)	10.7 (2.0) *^#$^	2.7 (0.3)	6.6 (1.4) *^&#^
Liver CRP (mg/mL)	93.9 (53.7)	64.3 (18.9)	76.7 (41.7)	60.3 (46.9)
Liver TNFα (mg/mL)	91.5 (47.8)	55.2 (49.3) *	85.2 (59.5)	54.5 (65.4) *

^×^ Adiposity index: (total abdominal fat × 100)/body weight. ^∆^ Hepatosomatic index: (liver weight × 100)/body weight. Results are means (standard deviation) (*n* = 7). * *P* < 0.05 vs. HFHS-C; ^&^ *P* < 0.05 vs. HFHS-FOM; ^#^ *P* < 0.05 vs. HFHS-GSE; ^$^ *P* < 0.05 vs. HFHS-FOM&GSE. Comparisons were performed using the one-way ANOVA and Tukey post hoc tests. These parameters have already been studied in the same cohort of animals and partially published [15,20,23]. ORAC: Oxygen Radical Absorbance Capacity; GPX: glutathione peroxidase; TFA: total fatty acids; FFA: free fatty acids.

**Table 2:** Biometric measurements, biochemical and oxidative stress parameters measured in rats fed standard (STD) diets. C: control; GSE: grape seed polyphenols extract; FOM: fish oil mixture.

	STD-C	STD-FOM	STD-GSE	STD-FOM&GSE
Weight Increase (%)	83.8 (22.1)	92.9 (21.3)	81.4 (11.6)	88.8 (23.2)
Adiposity index (%) ^×^	1.92 (0.48)	3.99 (1.20) *	6.17 (3.02) *	4.84 (1.19) *
Hepatosomatic index (%) ^∆^	2.6 (0.5)	2.7 (0.2)	2.7 (0.2)	2.7 (0.2)
Total cholesterol (mmol/L)	4.3 (1.0)	3.8 (0.2)	4.4 (0.3)	3.5 (0.4)
Triglycerides (mmol/L)	1.5 (0.4)	1.7 (0.4)	1.9 (0.3)	1.6 (0.3)
FFA (μg/mL)	212.0 (22.1)	164.2 (32.0)	166.6 (24.2)	147.5 (30.0) *
TFA (μg/mL)	2982.0 (772.8)	2873.8 (343.7)	3426.7 (338.5)	2770.9 (438.6)
HbA1c (%)	3.24 (0.15)	3.15 (0.10)	3.25 (0.22)	3.35 (0.20)
Fasting Glucose (mg/dL)	64.57 (2.70)	71.00 (6.53)	61.00 (3.37)	64.86 (5.87)
Plasma insulin (ng/mL)	0.9 (0.3)	1.2 (0.6)	1.2 (0.5)	1.5 (0.5)
ORAC (µmol Trolox/mL plasma)	17.6 (8.3)	18.8 (4.4)	16.6 (5.1)	22.1 (2.9)
Plasma GPX(U/g Hb)	0.4 (0.1)	1.2 (0.7) *^#$^	6.1 (0.5) *^&$^	19.2 (3.01) *^&#^
TBARS (mg MDA/kg liver)	4.5 (1.4)	10.0 (2.4) *^#^	3.2 (0.2 7) ^&$^	7.6 (1.7) ^#^
Liver CRP (mg/mL)	91.4 (21.6)	45.4 (15.7) *	49.7 (7.9) *	56.1 (26.5) *
Liver TNFα (mg/mL)	116.0 (72.5)	63.8 (26.3) *	73.1 (66.7) *	62.6 (47.6) *

^†^ Adiposity index: (total abdominal fat × 100)/body weight. ^∆^ Hepatosomatic index: (liver weight × 100)/body weight. Results are means (standard deviation) (*n* = 7). * *P* < 0.05 vs. STD-C; ^&^ *P* < 0.05 vs. STD-FOM; ^#^ *P* < 0.05 vs. STD-GSE; ^$^ *P* < 0.05 vs. STD-FOM&GSE. Comparisons were performed using the one-way ANOVA and Tukey post hoc tests. These parameters have already been studied in the same cohort of animals and partially published [15,19,23]. ORAC: Oxygen Radical Absorbance Capacity; GPX: glutathione peroxidase; TFA: total fatty acids; FFA: free fatty acids.

**Table 3:** Carbonylated proteins identified from 2-DE gels of both high-fat and high-sucrose (HFHS) (Figure 5) and standard (STD) (Figure 6) diets. Spots of interest were identified by LC-ESI-IT-MS/MS as described in the Materials and Methods Section. Spot N° refers to numbered spot on Figure 5 and Figure 6.

Spot *N*°	UniprotKB Accession	Protein Description	Gene Name	Subcellular Localization	Avg. Mass (Da)	Coverage (%)	#Peptides (#Unique)
1	P07756	Carbamoyl-phosphate synthase [ammonia] mitochondrial	Cps1	Mitochondria	164,579	70	73 (69)
2	P12346	Serotransferrin	Tf	Extracellular region or secreted	76,346	38	26 (26)
3	P02770	Serum albumin	Alb	Extracellular region or secreted	68,731	42	45 (45)
4	P50137	Transketolase	Tkt	Endoplasmic reticulum/Peroxisome	67,644	49	43 (43)
5,6	P04762	Catalase	Cat	Peroxisome	59,757	27	18 (18)
7	P63039	60 kDa heat shock protein mitochondrial	Hspd1	Mitochondria	60,956	31	21 (21)
8	P04785	Protein disulfide-isomerase	P4hb	Endoplasmic reticulum	56,951	38	25 (25)
9	P11884	Aldehyde dehydrogenase mitochondrial	Aldh2	Mitochondria	56,488	31	24 (24)
10	P85968	6-phosphogluconate dehydrogenase decarboxylating	Pgd	Mitochondria	53,236	20	10 (10)
11	P60711	Actin cytoplasmic 1	Actb	Cytoskeleton	41,737	56	49 (24)
12	P15650	Long-chain specific acyl-CoA dehydrogenase mitochondrial	Acadl	Mitochondria	47,873	36	24 (24)
	P32755	4-hydroxyphenylpyruvate dioxygenase	Hpd	Endoplasmic reticulum	45,112	45	24 (24)
	Q03248	Beta-ureidopropionase	Upb1	Cytoplasm	44,042	28	12 (12)
13	P09034	Argininosuccinate synthase	Ass1	Cytosol	46,496	54	48 (48)
14	P16617	Phosphoglycerate kinase 1	Pgk1	Cytoplasm	44,538	20	7 (7)
	P13437	3-ketoacyl-CoA thiolase mitochondrial	Acaa2	Mitochondria	41,871	41	19 (19)
15	P25093	Fumarylacetoacetase	Fah	Cytosol/Extracellular region or secreted	45,976	10	4 (4)
16	P06757	Alcohol dehydrogenase 1	Adh1	Cytoplasm	39,645	46	28 (24)
17	P00507	Aspartate aminotransferase mitochondrial	Got2	Mitochondria	47,314	43	31 (30)
18	P00481	Ornithine carbamoyltransferase mitochondrial	Otc	Mitochondria	39,886	27	9 (9)
19	Q03336	Regucalcin	Rgn	Cytoplasm	33,390	53	27 (27)
20	P23457	3-alpha-hydroxysteroid dehydrogenase	Akr1c9	Cytoplasm	37,028	40	20 (12)
21	Q497B0	Omega-amidase NIT2	Nit2	Cytoplasm	30,701	28	10 (10)

**Table 4:** Carbonylation index calculated for each protein in high-fat and high-sucrose (HFHS) diets. Spot N° refers to numbered spot on Figure 5.

			CARBONYLATION INDEX ^1^				FOLD CHANGE^2^		
Spot *N*°	Protein Description	Gene Name	HFHS-C	HFHS-FOM	HFHS-GSE	HFHS-FOM&GSE	HFHS-FOM/HFHS-C	HFHS-GSE/HFHS-C	HFHS-FOM&GSE/HFHS-C
1	Carbamoyl-phosphate synthase [ammonia] mitochondrial	Cps1	0.27 (0.01)	0.32 (0.14)	0.22 (0.11)	0.31 (0.06)	1.19	0.81	1.15
2	Serotransferrin	Tf	0.24 (0.05)	0.53 (0.25)	0.19 (0.10)	0.47 (0.04) *^#^	2.21	0.79	1.96
3	Serum albumin	Alb	1.18 (0.04)	0.50 (0.18) *^#^	1.33 (0.52) ^&^	0.66 (0.12) *	0.42	1.13	0.56
4	Transketolase	Tkt	0.64 (0.13)	0.67 (0.01)	0.52 (0.22)	0.53 (0.52)	1.05	0.81	0.83
5,6	Catalase	Cat	1.33 (0.43)	0.93 (0.50)	0.77 (0.14) *	0.57 (0.30) *	0.70	0.58	0.43
7	60 kDa heat shock protein mitochondrial	Hspd1	0.55 (0.07)	0.60 (0.12)	1.45 (0.82)	0.31 (0.03) *^&^	1.09	2.64	0.56
8	Protein disulfide-isomerase	P4hb	0.76 (0.15)	0.85 (0.08)	0.98 (3.42)	0.93 (0.62)	1.12	1.29	1.22
9	Aldehyde dehydrogenase mitochondrial	Aldh2	0.61 (0.12)	0.17 (0.08) *^#^	2.38 (0.26) *^&$^	0.54 (0.23) ^#^	0.28	3.90	0.89
10	6-phosphogluconate dehydrogenase decarboxylating	Pgd	1.01 (0.02)	3.98 (1.20) *^$^	1.31 (1.20)	0.35 (0.01) *^&^	3.94	1.30	0.35
11	Actin cytoplasmic 1	Actb	0.11 (0.05)	0.43 (0.14) *^#^	0.15 (0.09)	0.69 (0.27) *^#^	3.91	1.36	6.27
12	Long-chain specific acyl-CoA dehydrogenase mitochondrial	Acadl	1.89 (0.35)	0.99 (0.18) *^$^	0.82 (0.13) *^$^	0.37 (0.01) *^&#^	0.52	0.43	0.20
	4-hydroxyphenylpyruvate dioxygenase	Hpd							
	Beta-ureidopropionase	Upb1							
13	Argininosuccinate synthase	Ass1	0.75 (0.42)	0.42 (0.13)	0.35 (0.17)	0.25 (0.26)	0.56	0.47	0.33
14	Phosphoglycerate kinase 1 3-ketoacyl-CoA thiolase mitochondrial	Pgk1 Acaa2	0.22 (0.10)	0.33 (0.09)	0.13 (0.12)	0.36 (0.01)	1.50	0.59	1.64
15	Fumarylacetoacetase	Fah	0.80 (0.76)	0.55 (0.37)	0.55 (0.25)	0.39 (0.01)	0.69	0.69	0.49
16	Alcohol dehydrogenase 1	Adh1	1.21 (0.14)	1.69 (0.98)	1.55 (0.55)	1.23 (0.21)	1.40	1.28	1.02
17	Aspartate aminotransferase mitochondrial	Got2	1.41 (0.61)	1.01 (0.89)	4.46 (0.94) *^&^	2.63 (1.02)	0.72	3.16	1.87
18	Ornithine carbamoyltransferase mitochondrial	Otc	0.86 (0.18)	0.61 (0.06) *^$^	0.26 (0.22) *	0.32 (0.01) *^&^	0.71	0.30	0.37
19	Regucalcin	Rgn	0.18 (0.25)	0.29 (0.13)	0.51 (0.23)	0.29 (0.15)	1.61	2.83	1.61
20	3-alpha-hydroxysteroid dehydrogenase	Akr1c9	0.20 (0.07)	0.19 (0.14)	0.55 (0.15) *^&^	0.30 (0.23)	0.95	2.75	1.50
21	Omega-amidase NIT2	Nit2	1.88 (0.29)	4.76 (1.20) *^#$^	1.76 (0.18) ^&$^	0.79 (0.01) *^&#^	2.53	0.94	0.42

^1^ Results are means (standard deviation) obtained from the densitometric analysis of the 2-DE gels represented in Figure 5 and Figure 6. Data (*n* = 3) correspond to three independent 2-DE experiments performed in triplicates. * *P* < 0.05 vs. HFHS-C; ^&^ *P* < 0.05 vs. HFHS-FOM; ^#^ *P* < 0.05 vs. HFHS-GSE; ^$^ *P* < 0.05 vs. HFHS-FOM&GSE. Comparisons were performed using the one-way ANOVA and Tukey post hoc tests. ^2^ Fold change of carbonylation index in comparison to HFHS-C. Green color represents a significant (*P* < 0.05) decrease in the carbonylation level and red color represents significant (*P* < 0.05) increases. C: control; GSE: grape seed polyphenols extract; FOM: fish oil mixture.

**Table 5:** Carbonylation index calculated for each protein in standard (STD) diets. Spot N° refers to numbered spot on Figure 6.

			CARBONYLATION INDEX ^1^				FOLD CHANGE ^2^		
Spot *N*°	Protein Description	Gene Name	STD-C	STD-FOM	STD-GSE	STD-FOM&GSE	STD-FOM/STD-C	STD-GSE/STD-C	STD-FOM&GSE/STD-C
1	Carbamoyl-phosphate synthase [ammonia] mitochondrial	Cps1	0.15 (0.14)	0.11 (0.01)	0.20 (0.11)	0.19 (0.07)	0.73	1.33	1.27
2	Serotransferrin	Tf	0.13 (0.06)	0.08 (0.07)	0.09 (0.08)	0.19 (0.09)	0.62	0.69	1.46
3	Serum albumin	Alb	0.50 (0.03)	0.20 (0.08) *^#^	0.62 (0.22)	0.40 (0.16)	0.40	1.24	0.80
4	Transketolase	Tkt	0.61 (0.32)	0.39 (0.09)	0.91 (0.21) ^&$^	0.43 (0.10)	0.64	1.49	0.70
5,6	Catalase	Cat	0.72 (0.16)	0.96 (0.08)	1.63 (0.46) *^&$^	1.05 (0.14)	1.33	2.26	1.46
7	60 kDa heat shock protein mitochondrial	Hspd1	0.45 (0.25)	0.55 (0.22)	0.17 (0.08) ^&$^	0.88 (0.27)	1.22	0.38	1.96
8	Protein disulfide-isomerase	P4hb	0.60 (0.16)	2.68 (0.79) *^#$^	0.77 (0.07) ^&$^	1.37 (0.45) *^&#^	4.47	1.28	2.28
9	Aldehyde dehydrogenase mitochondrial	Aldh2	3.84 (0.54)	5.43 (1.15)	4.73 (0.97)	6.51 (0.95) *	1.41	1.23	1.70
10	6-phosphogluconate dehydrogenase decarboxylating	Pgd	1.84 (0.16)	1.55 (0.73)	1.01 (0.27) *	1.41 (0.14)	0.84	0.55	0.77
11	Actin cytoplasmic 1	Actb	0.19 (0.10)	0.28 (0.09)	0.42 (0.06) *^&^	0.49 (0.04) *^&^	1.47	2.21	2.58
12	Long-chain specific acyl-CoA dehydrogenase mitochondrial	Acadl	0.90 (0.54)	2.02 (0.87)	1.25 (0.27)	0.46 (0.07) ^&#^	2.24	1.39	0.51
	4-hydroxyphenylpyruvate dioxygenase	Hpd							
	Beta-ureidopropionase	Upb1							
13	Argininosuccinate synthase	Ass1	0.23 (0.06)	0.21 (0.04) ^$^	0.36 (0.03) *^$^	0.50 (0.04) *^&#^	0.91	1.57	2.17
14	Phosphoglycerate kinase 1 3-ketoacyl-CoA thiolase mitochondrial	Pgk1 Acaa2	0.31 (0.07)	0.24 (0.11)	0.27 (0.10)	0.42 (0.38)	0.77	0.87	1.35
15	Fumarylacetoacetase	Fah	0.90 (0.80)	0.89 (0.23) ^#$^	1.47 (0.21) ^&$^	2.23 (0.13) *^&#^	0.99	1.63	2.48
16	Alcohol dehydrogenase 1	Adh1	0.79 (0.11)	0.62 (0.12)	1.03 (0.22) ^&$^	0.58 (0.21)	0.78	1.30	0.73
17	Aspartate aminotransferase mitochondrial	Got2	0.23 (0.06)	0.13 (0.01) *^#$^	0.47 (0.00) *^&^	0.54 (0.12) *^&^	0.57	2.04	2.35
18	Ornithine carbamoyltransferase mitochondrial	Otc	0.69 (1.16)	0.70 (0.13) ^#$^	0.38 (0.11) ^&$^	1.56 (0.15) ^&#^	1.01	0.55	2.26
19	Regucalcin	Rgn	0.28 (0.09)	0.60 (0.27)	0.19 (0.21)	0.38 (0.19)	2.14	0.68	1.36
20	3-alpha-hydroxysteroid dehydrogenase	Akr1c9	0.85 (0.14)	0.97 (0.22) ^#^	0.65 (0.09) *^&^	0.76 (0.19)	1.14	0.76	0.89
21	Omega-amidase NIT2	Nit2	4.84 (2.42)	2.60 (0.34) *^#^	3.82 (0.44) *	2.68 (0.32) *^#^	0.54	0.79	0.55

^1^ Results are means (standard deviation) obtained from the densitometric analysis of the 2-DE gels represented in Figure 5 and Figure 6. Data (*n* = 3) correspond to three independent 2-DE experiments performed in triplicates. * *P* < 0.05 vs. STD-C; ^&^ *P* < 0.05 vs. STD-FOM; ^#^ *P* < 0.05 vs. STD-GSE; ^$^ *P* < 0.05 vs. STD-FOM&GSE. Comparisons were performed using the one-way ANOVA and Tukey post hoc tests. ^2^ Fold change of carbonylation index in comparison to HFHS-C. Green color represents a significant (*P* < 0.05) decrease in the carbonylation level and red color represents significant (*P* < 0.05) increases. C: control; GSE: grape seed polyphenols extract; FOM: fish oil mixture.

## Supplementary Materials

The following are available online at https://www.mdpi.com/1660-3397/18/1/34/s1, Table S1: Detailed pathways and processes enriched in the rat liver protein carbonylome by FOM, GSE and FOM&GSE in HFHS and STD diets analyzed by Metascape tool. Figure S1: Linear range and standard curve for albumin quantification based on the Coomassie intensity of rat plasma samples resolved in 1-D SDS-PAGE.
