# Supplementary material for: Modulation of the Liver Protein Carbonylome by the Combined Effect of Marine Omega-3 PUFAs and Grape Polyphenols Supplementation in Rats Fed an Obesogenic High Fat and High Sucrose Diet
Source: Mar Drugs. 2019 Dec 30;18(1):34. doi: 10.3390/md18010034 (PMC7024381; doi:10.3390/md18010034)
Supplement: Supplementary file 1 [file marinedrugs-18-00034-s001.pdf]

Supplementary

Modulation of the Liver Protein Carbonylome by the Combined Effect of Marine Omega-3 PUFAs and Grape Polyphenols Supplementation in Rats Fed an Obesogenic High Fat and High Sucrose Diet

Lucía Méndez [1,*], Silvia Muñoz [1], Bernat Miralles-Pérez [2], M. Rosa Nogués [2], Sara Ramos-Romero [3,4], Josep L. Torres [3] and Isabel Medina [1]

Figure S1. Determination of the linear range for the quantification of albumin in rat plasma base on Coomassie intensity. Bands were analyzed by using LabImage 1D (Kapelan Bio-Imaging Solutions, Halle, Germany).

Rat plasma (μg)	Coomassie intensity for Albumin
2	18230
5	40903
7	91847
10	138208
15	213675
20	257536
25	321583
30	265843

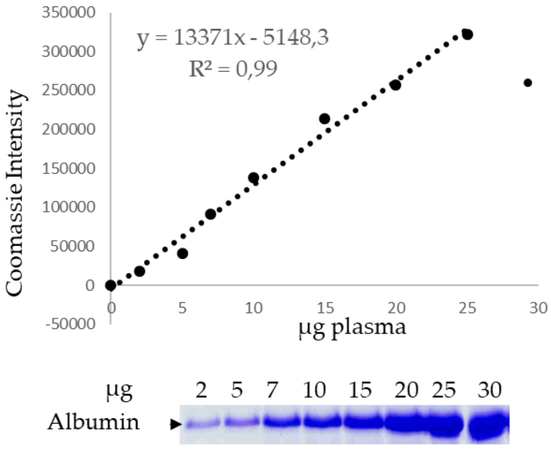

Supplementary Table S1. Pathways and processes enriched by FOM, GSE and FOM&GSE in rats fed either HFHS or STD diet. "Count" is the number of genes with membership in the given ontology term. "%" is the percentage of all of the user-provided genes that are found in the given ontology term (only input genes with at least one ontology term annotation are included in the calculation). "Log10(P)" is the p-value in log base 10. "Log10(q)" is the multi-test adjusted p-value in log base 10.[1]. HFHS: high-fat and high-sucrose diet; STD: standard diet; GSE: grape seed polyphenols extract; FOM: fish oil mixture.

Background diet	Supplement	GO	Category	Description	Count	%	Log10(P)	Log10(q)	Gene Symbols	Other significant members in the cluster (in order of significance)
					**HFHS DIETS**					
HFHS	FOM	GO:0044282	GO Biological Processes	small molecule catabolic process	5	50.00	-6.32	-2.06	Acadl, Otc, Hpd, Aldh2, Upb1, Cat, Pgd	protein homooligomerization; organic acid catabolic process; carboxylic acid catabolic process; protein complex oligomerization; liver development; hepaticobiliary system development; small molecule biosynthetic process; carboxylic acid biosynthetic process; organic acid biosynthetic process; gland development
HFHS	FOM	GO:0006520	GO Biological Processes	cellular amino acid metabolic process	4	40.00	-4.97	-1.01	Otc, Hpd, Upb1, Nit2	alpha-amino acid metabolic process
HFHS	FOM	GO:0042737	GO Biological Processes	drug catabolic process	3	30.00	-4.18	-0.52	Cat, Hpd, Aldh2, Acadl, Otc, Actb	alcohol metabolic process, organic hydroxy compound metabolic process, response to acid chemical, response to toxic substance, drug metabolic process
HFHS	FOM	GO:0007584	GO Biological Processes	response to nutrient	3	30.00	-3.05	0.00	Alb, Cat, Otc	response to metal ion, response to nutrient levels, response to extracellular stimulus, response to inorganic substance

[1]Gene names in green colour represent proteins that decreased their carbonylation level by the supplement and gene names in red colour represent proteins that increased their carbonylation level by the supplement

HFHS	FOM	GO:0032787	GO Biological Processes	monocarboxylic acid metabolic process	3	30.00	-2.52	0.00	Acadl, Nit2, Pgd	
HFHS	FOM	R-RNO-168249	Reactome Gene Sets	Innate Immune System	3	30.00	-2.21	0.00	Cat, Actb, Nit2	
HFHS	GSE	GO:0044282	GO Biological Processes	small molecule catabolic process	6	75.00	-9.00	-4.75	Acadl, Otc, Got2, Hpd, Aldh2, Upb1	organic acid catabolic process, carboxylic acid catabolic process, cellular amino acid biosynthetic process, cellular amino acid metabolic process, alpha-amino acid catabolic process, cellular amino acid catabolic process, carboxylic acid biosynthetic process<br>organic acid biosynthetic process, Metabolism of amino acids and derivatives, alpha-amino acid metabolic process, small molecule biosynthetic process
HFHS	GSE	GO:0006066	GO Biological Processes	alcohol metabolic process	4	50.00	-5.21	-1.80	Cat, Acadl, Aldh2, Akr1c9, Hpd	drug catabolic process, antibiotic metabolic process, organic hydroxy compound metabolic process, steroid metabolic process, drug metabolic process
HFHS	GSE	GO:0051260	GO Biological Processes	protein homooligomerization	4	50.00	-4.86	-1.65	Cat, Acadl, Otc, Upb1	protein complex oligomerization
HFHS	GSE	GO:0042180	GO Biological Processes	cellular ketone metabolic process	3	37.50	-4.17	-1.12	Cat, Got2, Akr1c9	cofactor metabolic process
HFHS	GSE	GO:0048732	GO Biological Processes	gland development	4	50.00	-4.01	-1.03	Otc, Got2, Aldh2, Upb1	liver development, hepaticobiliary system development

HFHS	GSE	GO:0032868	GO Biological Processes	response to insulin	3	37.50	-3.53	-0.67	Cat, Otc, Got2, Aldh2	response to acid chemical; response to peptide hormone; response to peptide; response to toxic substance
HFHS	FOM&GSE	GO:0006520	GO Biological Processes	cellular amino acid metabolic process	4	36.36	-4.78	-0.65	Otc, Hpd, Upb1, Nit2	alpha-amino acid metabolic process
HFHS	FOM&GSE	GO:0044282	GO Biological Processes	small molecule catabolic process	4	36.36	-4.48	-0.65	Acadl, Otc, Hpd, Upb1, Cat, Pgd	protein homooligomerization, organic acid catabolic process, carboxylic acid catabolic process, protein complex oligomerization, small molecule biosynthetic process, carboxylic acid biosynthetic process, organic acid biosynthetic process
HFHS	FOM&GSE	GO:0007584	GO Biological Processes	response to nutrient	4	36.36	-4.31	-0.65	Alb, Cat, Otc, Hspd1, Tf, Actb	response to inorganic substance, response to metal ion, response to nutrient levels, response to extracellular stimulus, response to hypoxia, response to decreased oxygen levels, response to oxygen levels, response to toxic substance
HFHS	FOM&GSE	R-RNO-5653656	Reactome Gene Sets	Vesicle-mediated transport	3	27.27	-2.55	0.00	Alb, Tf, Actb	
HFHS	FOM&GSE	GO:0032787	GO Biological Processes	monocarboxylic acid metabolic process	3	27.27	-2.39	0.00	Acadl, Nit2, Pgd	
HFHS	FOM&GSE	R-RNO-168249	Reactome Gene Sets	Innate Immune System	3	27.27	-2.08	0.00	Cat, Actb, Nit2	
**STANDARD DIETS**										
STD	FOM	No significant pathways/process enriched								

STD	GSE	rno01200	KEGG Pathway	Carbon metabolism	4	40.00	-6.38	-2.12	Cat, Got2, Tkt, Pgd, Akr1c9	cofactor metabolic process
STD	GSE	GO:0006081	GO Biological Processes	cellular aldehyde metabolic process	3	30.00	-5.18	-1.45	Adh1, Tkt, Pgd, Akr1c9, Nit2	monocarboxylic acid metabolic process
STD	GSE	GO:0009064	GO Biological Processes	glutamine family amino acid metabolic process	3	30.00	-5.11	-1.45	Otc, Got2, Nit2, Tkt, Adh1, Pgd	glutamine family amino acid metabolic process; biosynthesis of amino acids, alpha-amino acid metabolic process, cellular amino acid metabolic process, small molecule catabolic process, small molecule biosynthetic process
STD	GSE	GO:0009636	GO Biological Processes	response to toxic substance	5	50.00	-4.66	-1.17	Adh1, Cat, Got2, Hspd1, Actb, Akr1c14	response to activity, antibiotic metabolic process, response to antibiotic, response to ethanol, alcohol metabolic process, response to alcohol, organic hydroxy compound metabolic process, drug metabolic process
STD	GSE	GO:0032868	GO Biological Processes	response to insulin	3	30.00	-3.21	-0.13	Cat, Otc, Got2, Hspd1, Adh1	response to insulin, response to nutrient, response to acid chemical, response to peptide hormone, response to peptide, response to nutrient levels, response to extracellular stimulus, response to inorganic substance
STD	GSE	R-RNO-168249	Reactome Gene Sets	Innate Immune System	3	30.00	-2.21	0.00	Cat, Actb, Nit2	
STD	FOM&GSE	GO:1901605	GO Biological Processes	alpha-amino acid metabolic process	5	62.50	-7.98	-3.79	P4hb, Otc, Ass1, Got2,	glutamine family amino acid metabolic process; cellular amino acid metabolic process; arginine metabolic process; alpha-amino

									Fah, Aldh2	acid catabolic process; small molecule catabolic process; cellular amino acid catabolic process; organic acid catabolic process; carboxylic acid catabolic process
STD	FOM&GSE	rno00220	KEGG Pathway	Arginine biosynthesis	3	37.50	-7.19	-3.63	Otc, Ass1, Got2, Cat, Aldh2	alpha-amino acid biosynthetic process; cellular amino acid biosynthetic process; Biosynthesis of amino acids; Metabolism of amino acids and derivatives; response to acid chemical; response to peptide hormone; gland development; response to peptide; liver development; hepatobiliary system development; response to insulin; carboxylic acid biosynthetic process; organic acid biosynthetic process; response to nutrient; response to metal ion; small molecule biosynthetic process; response to nutrient levels; response to extracellular stimulus; response to inorganic substance
STD	FOM&GSE	GO:0009636	GO Biological Processes	response to toxic substance	5	62.50	-5.28	-1.97	Cat, Ass1, Got2, Aldh2, Actb	
STD	FOM&GSE	GO:0070542	GO Biological Processes	response to fatty acid	3	37.50	-4.72	-1.58	Cat, Ass1, Aldh2, Fah, P4hb	drug catabolic process; response to estradiol; drug metabolic process; organic hydroxy compound metabolic process; response to oxygen levels
